# The Faith Relationship Interview for Adolescents: A Qualitative Explorative Study in the Netherlands to Evaluate a New Method to Integrate Religion in Psychotherapy

**DOI:** 10.1007/s10943-025-02389-1

**Published:** 2025-07-28

**Authors:** Sanne G. Helder, Gerda Mosterd-Pol, Marieke van der Maten, Hanneke Schaap-Jonker

**Affiliations:** 1 Centre for Research and Innovation in Christian Mental Health Care, Hoevelaken, The Netherlands; 2 Eleos Mental Health Care, Hoevelaken, The Netherlands; 3 Faculty of Social Sciences and Humanities, School of Religion and Theology, Vrije Universiteit, De Boelelaan 1105, 1081 HV Amsterdam, The Netherlands

**Keywords:** Faith relationship interview, God representations, Intervention, Psychotherapy, Religion

## Abstract

The faith relationship interview (FRI) has been recently developed as an intervention in which clients discuss their religious thoughts and feelings, their (development of the) God representations, and the parallels and differences between their relationship with God and with other people. In this explorative qualitative research, the FRI is evaluated with eight adolescent clients and four clinicians in the Netherlands. All participants reported to have positive experiences regarding the FRI and clients reported to have learned something about their God representations and about the parallel between their relationships with God and others. These results suggest that the FRI is a promising and valuable intervention for Christian clients and clinicians to integrate religion and God representations in psychotherapy.

## Introduction

Research has demonstrated that religious psychiatric clients often experience religious or spiritual struggles and a negative God representation (Schaap-Jonker et al., 2002, 2017; Van Nieuw Amerongen-Meeuse et al., 2022) and that integrating religion and spirituality in mental healthcare can lead to a more effective therapy (AbdAleati et al., 2016; Bouwhuis-Van Keulen et al., 2023; Captari et al., 2018; Gonçalves et al., 2015; Paukert et al., 2011). Integration appears important, particularly when religion and spirituality are part of psychological suffering (Mosterd-Pol et al., 2021; Pargament & Exline, 2022), or when clients express a desire to discuss these topics in their therapy (Sandage et al., 2020). The question remaining, however, is how this integration can be realized. In this article, the faith relationship interview (FRI; in Dutch: Geloofsrelatiegesprek (GRG), a new diagnostic method that integrates religious struggles and God representations in psychotherapy, developed in the Netherlands, will be evaluated.

Different methods have been developed for the integration of the clients’ religion and spirituality in therapy, for example the religious anamnesis (Glas, 2009; Pargament & Exline, 2022). However, these interview questions neither focus on the God relationship/God representation as a core element in religious/spiritual life, nor are the interrelationships of representations of God, self and others reflected in the questions. From a psychological and clinical perspective, the latter is crucial to gain deeper insight into relational dynamics in religious life and/or spiritual struggles and into the functioning of R/S in the context of life history and current psychopathology. Therefore, Mosterd-Pol et al. (2021) developed the FRI as a new method to address these issues.

The FRI discusses the clients’ religious thoughts and feelings, and their (development of the) God representations. As these are highly personal and contextual topics, clients and therapists might experience difficulties talking about these in therapy, feeling that they are not on the same level concerning religion and spirituality. This so-called religiosity gap might lead to negative experiences for clients, for example discomfort and disrespect, while a religiosity match is perceived positively (Van Nieuw Amerongen-Meeuse et al., 2018). The FRI aims to bridge this religiosity gap by encouraging the client to share their unique religious narrative from a psychological point of view in a structured way. The therapist listens and seeks to understand the psychological dynamics of the client’s worldview instead of theological beliefs. By centering the conversation on the client’s religious experiences and the development of God representations, the FRI seeks to prevent negative experiences stemming from a religiosity gap, fostering a more empathetic and collaborative therapeutic relationship.

In an explorative study of Mosterd-Pol et al. (2021) 23 adult clients with depressive disorders were included to evaluate the FRI. They reported that (1) the FRI has led to more insight into their God representations and that (2) they felt more understood by their therapist, reflecting a religiosity match. Obviously, this method needs more evaluation. Interestingly, youth therapists have also shown interest in the FRI, suggesting its potential relevance across different age groups. Overall, most research on God representations and the integration of religion and spirituality in clinical therapy has been done among adults (Dew et al., 2008). Hence, there is a need for research with a focus on adolescents, since adolescents are in the process of forming their (religious) identity. Furthermore, aspects of God representations seem to be related to their mental well-being, with self-worth being associated with more positive, loving God representations and less negative, rejecting ones (Francis et al., 2001). Therefore, the current article evaluates the FRI among adolescents, ages 15–19, in clinical therapy.

### The Faith Relationship Interview

The base of the FRI is the Questionnaire of God Representations, which addresses both experiential and doctrinal representations of God (Schaap-Jonker et al., 2008, 2016; Sharp et al., 2021). In a clinical context, this instrument seems to tap not only explicit, but also more implicit aspects of God representations (Stulp et al., 2019). The Interpersonal Psychotherapy (IPT) functions as a guiding principle for the FRI. Developed by Klerman, Weissman and colleagues in 1974, IPT focusses on the experiences of stressful life events as loss, life changes or disputes and accentuates the importance of relationships in recovery (Klerman et al., 1993; Ravitz et al., 2019). In the diagnostic phase of IPT, the clinician asks clients about their feelings, thoughts and behaviors related to particular relationships, examining similar experiences in various relationships.

In the FRI, clients are asked about their God representations and their development, representations of self and representation of others. According to the theory about God representations formulated by Rizzuto (1979), God representations are developed in early, nurturing relationships with significant others, such as parents or grandparents, and are unique for every individual, even when someone does not believe in God. Since the FRI discusses the God representations in the context of parent representations and self-representations of a client, it gives the clinician diagnostic information about the client’s psychological functioning. In this context, attention is paid to religious or spiritual struggles, which are frequently characterized by feelings such as anger toward and/or fear of God, disappointment, abandonment by God and a lack of positive emotions (Van Nieuw Amerongen-Meeuse et al., 2022). By addressing these struggles and relating them to interpersonal experiences, the FRI gives insight into how religion and spirituality are intertwined with psychological and relational issues. In addition, the client is explicitly asked for their wish on how to integrate religion and spirituality in therapy.

As the FRI focuses on the relational dimension of religion, in line with a relational spirituality model (Sandage & Brown, 2018), it enables the therapist to leave behind their own feelings and thoughts concerning religion. Since the interview is about the subjective experience of the client, a religiosity gap might not be experienced by clients (Mosterd-Pol et al., 2021). Table 1 describes the various steps of the FRI.

**Table 1:** Explanation steps of the FRI for adolescents

Explanation steps of the FRI for adolescents:	
1. At the start, the clinician and client agree upon how to address God, whereafter the clinician is able to match the way of addressing the client is familiar with, for example Lord, God, Lord God, Yahweh. Then, the clinician explains the structure of the rest of the interview	
2. The clinician shows the client the results of the Questionnaire of God Representations, explains the results and asks for recognition of the client. A question that could be asked is: “Compared to the norm group, you score low on positive feelings when you think about God, do you recognize that?”	
3. The meaning of the scales of the Questionnaire of God Representations for the client is explored. The clinician can do this by asking questions to recent feelings of the client toward God and the beliefs. An example question is: “You just mentioned that you experience positive feelings when you think about God. Did you experience those feelings recently? Could you share more about that?” The goal for the clinician is to get insights in the client’s life rules, conditional and core beliefs, and integration of feelings toward God	
4. The client’s development of the God representations during childhood, puberty and later life will be asked. A question that can be asked is: “How you feel about God at the moment, has it always been like this?”	
5. The clinician asks the client about the parallels and differences in interpersonal relationships and the relationship with God. Sample question: “The feelings and thoughts we discuss now in relation to God you mentioned before [step 4], do you recognize those in relation to other people?”	
6. The wish of the client to integrate faith in therapy or pastoral care is asked by the clinician	
7. The clinician tells the client that a report will be made that will be discussed in the next session	

### Aim and Relevance

To evaluate whether clients and clinicians perceive the FRI as a potential added value in their therapy, the following research question was formulated: ‘How is the use of the Faith Relationship Interview in Christian mental health care experienced and evaluated by clients between 15 and19 years old and their clinicians?’ Three specific objectives were: (1) to assess the opinions of the clients and clinicians regarding the faith relationship interviews, (2) to assess the interview’s contribution to new insights about faith relationships, (3) and to explore its added value for religion into therapy. This study will contribute to academic literature on the integration of religion and spirituality in clinical therapy for adolescents, as most studies on this topic, demonstrating that the incorporation of religion and spirituality in therapy might help the process of clinical recovery, were done among adults with mental health problems (Captari et al., 2018). Moreover, it contributes to the evaluation of the FRI, as this diagnostic method has only been researched once. Hence, both secular and religious institutes for mental health care, and thus society in general, could benefit from this research.

## Methodology

### Participants

Over a 3-month period, from March to May 2022, eight clients receiving therapy at the Dutch Christian mental health organization Eleos, were recruited. Informed consent was obtained from all clients and, if they were under 16 years old, from their parents as well. All clients were female, with an average age of 17 years at the time of the FRI (range: 15–19, SD: 1.3). Half of the clients belonged to a Strict Reformed denomination (4 out of 8). One client was at the moment of this research not a member of a church, but she and her family were guest members of a Strict Reformed congregation. This participant is therefore assigned to this denomination. Other information regarding denomination, primary disorder diagnoses and comorbidities can be found in Table 2.

**Table 2:** Client Demographic Characteristics

Client characteristic	*N*	*%*
Gender		
Male	0	0
Female	8	100
Denomination		
Strict Reformed	5	62.5
Mainstream Protestant	2	25
Evangelical	1	12.5
Primary disorder		
Adjustment disorder	1	12.5
Depressive disorder	2	25
Obsessive–compulsive disorder	1	12.5
Other personality disorder	1	12.5
Other specified anxiety disorder	1	12.5
Separation anxiety disorder	1	12.5
Unspecified personality disorder	1	12.5
Comorbidity included:		
Anorexia nervosa		
Attention deficit/hyperactivity disorder		
Depressive disorder		
Panic disorder		
Parent–child struggles		
Posttraumatic stress disorder		
Social anxiety disorder		

*N* = 8

The four clinicians participating in this research had different functions within Eleos: a healthcare psychologist in training to become a psychotherapist, a healthcare psychologist in training to become a clinical psychologist, a clinical psychologist and a psychotherapist. All clinicians were female.

### Procedure

This research has been approved by the Science and Ethics Committee of the Faculty of Religion and Theology of the Vrije Universiteit Amsterdam (VU) and by the internal committee of Eleos institute for mental health care.

After obtaining the approval of the ethical committees, one clinician at Eleos asked her colleagues to participate in this research and those colleagues asked some of their clients to participate. Clients who were interested in participating were given the information and informed consent letter. All clinicians read the guidebook on the FRI that has been modified for youth, took the online module and supervision and practiced the FRI with their colleagues. After obtaining the clients’ informed consent and if needed of their parents, an appointment with the clinician was made for the FRI and the session afterward to discuss the results of the FRI. Another appointment was made for the clients and the researcher to evaluate the FRI.

A few days before the planned FRI, the researcher sent an e-mail to the client with the Questionnaire of God Representations to be filled in online. The outcomes of the questionnaire were analyzed by the researcher and sent to the clinician prior to the FRI. The FRI was conducted during an appointment of 75 min. In the next appointment, feedback was given to the client by the clinician about the FRI. Within a week after this second appointment, the researcher interviewed the clients about their experiences of the FRI. After all FRI’s with the clients, the researcher interviewed all four clinicians as well. In the evaluation interviews, in the form of one-on-one semi-structured interviews, the researcher asked all clients and clinicians for their permission to record the interview. After the evaluation interviews, the clients were given a voucher of five euros.

The average time for the clients between the FRI and the session with their clinician to discuss the outcomes of the FRI, was 12 days (range: 7–21, SD: 4.6). After the session with their clinicians, the interview was planned for this research. The average time between the session with the clinician and the interview with the researcher was 4.6 days (range: 0–11, SD: 3.6). Four of the interviews with the researcher took place online and the other four took place at a location of Eleos, by choice of the clients. No differences were observed regarding the content between the online and offline interviews. All of the interviews were recorded and the recordings lasted on average for 22 min. After the interviews with the clients, the clinicians were interviewed. These interviews took place online and the recordings lasted on average for 53 min.

### Measurement Instruments

The Questionnaire of God Representations has two dimensions, namely the feelings experienced by someone in relation to God and the beliefs someone has about the actions and power of God. The first dimension, “feelings,” consists of 18 questions and three scales, namely “positive feelings,” “anxiety” and “anger.” The second dimension, “beliefs,” has 16 questions and three scales: “supportive actions,” “ruling and/or punishing actions,” and “passivity” (Murken, 1998; Schaap-Jonker & Eurelings-Bontekoe, 2009; Schaap-Jonker et al., 2008, 2016). In total, the questionnaire consists of 34 questions and answers are scored on a five-point scale, ranging from not at all applicable (1) to completely applicable (5). For this research, the questionnaire has been modified for youth by facilitating the formulation of the questions for youth, for example “I experience fear of being punished” is changed into “I am afraid of punishment,” and “God lets everything run its course” is changed into “God does not interfere in the world.” Since there are no normative data available specifically for youth, data of the clients were compared to the available normative data for the general population and for respondents of the religious denominations the clients were affiliated with.

To evaluate the opinions of the clients and clinicians of the FRI and analyze whether the interview has given new insights in the God representations, self-representations, relationships with others and request for help of the clients, interview questions were drafted by the researcher and supervisor for the interviews.

### Data analysis

Before the FRI with their clinicians, all clients filled in the Questionnaire of God Representations online. Data were analyzed using statistical analysis software (SPSS 27). There were no missing data.

The recordings of the evaluation interviews with all clients and clinicians were transcribed and analyzed. The researcher utilized a qualitative thematic analysis method as described by Bryman (2016). The answers given in the interviews were coded on the basis of four main subjects, namely (1) positive and/or negative experience, (2) insight in self-representations, God representations, relationships with others and request for help, (3) the experience of religiosity gap, and (4) the added value for the FRI in therapy. To enhance the reliability of the analysis, the transcript of the first interview was analyzed by an independent coder. Their coding was then compared to the analysis of the researcher to examine whether the two analyses matched each other. The two analyses showed strong agreement (similarity of 7 of the 8 codes), confirming the validity of the coding scheme. Based on this confirmation, the researcher proceeded to code the remaining interviews independently.

## Results

### Results of the Opinions of Clients and Clinicians of the FRI

#### Opinions of Clients

Clients were asked to share their thoughts of the FRI. The qualitative thematic analysis identified several key themes regarding their experiences, which were further quantified to show the prevalence of each theme (Table 3).

**Table 3:** Opinions of Clients on the FRI

Item	Answer	Frequency (N = 8)	Example of answer
Positive experience	Yes No	8 0	“I thought it was very pleasant and interesting” (P6)
Negative experience	Yes No	2 0	“It took a long time” (P1) “I did not have any negative feelings” (P8)
Could anyone learn from the interview?	Yes No	7 0	“It could be very helpful for a lot of people to gain insight in their faith” (P6)
Experience of religiosity gap	Yes No	1 7	“The clinician encouraged me to see it differently. It made me think and I am still not sure how I feel about that” (P8) “No, I did not experience any difference” (P1)

All eight participants mentioned positive experiences regarding the structure and the outcomes of the FRI. Positive feelings were reported on the purpose of the interview to discuss religion in therapy (Participant 3, P7) and on the structure and manner of the interview (P1, P5, P7): that it was a respectful interview in which there was enough time to share the religious experiences and life stories without judgment of the clinician.

“It could be very helpful for a lot of people to gain insight in their faith.” (P6).

For almost half of the clients, it was pleasant to be able to read and discuss the report of the interview and change or explain parts of it (P4, P6, P7). Almost all clients reported to be positive about the interesting insights and awareness they gained during the interview (P3–P8). Two clients (P1 and P6) mentioned negative aspects as well: The FRI was taking too long, and it was intense and tiresome. For one of them the purpose of the interview was not entirely clear (P6?). The average grade for the FRI given by the clients was 8/10 (min. 1, max. 10, range 7.5–8.5, N = 7).

Three clients gave a recommendation for the FRI. One client shared that she would have appreciated it if her clinician would have shared more about one’s own vision on religion so that she could learn from this (P6). Two other clients wished they had the interview earlier in their therapy (P3, P7). Other recommendations given by clients were to have more time for the FRI (P7) and to specify the purpose of the interview and of the session after the FRI (P3, P6).

According to all clients asked, everyone could learn something of the FRI (P1–8). The most common answers on why others could learn from the interview were that it has given insight and awareness of one’s own faith, God representations, and the development of the God representations.

Seven clients did not experience a religiosity gap between them and their clinician (P1–P7). Six of them evaluated this as a positive experience and the other client found it to be strange that the clinician did not share their own religion and only asked questions about the client’s faith (P2). Later, the same client shared that the most important thing for her during the FRI was that the clinician asked questions to make sure the client was being understood. One client did experience a religiosity gap, yet the client did not experience this in a negative way (P8). For her, it was important for the clinician to open up in order for her to feel comfortable to talk about religion and faith.

### Opinions of Clinicians

The clinicians were asked the share their thoughts of the FRI as well. The clinicians’ responses were analyzed similarly using thematic coding, with themes identified and quantified where relevant (Table 4).

**Table 4:** Opinions of Clinicians on the FRI

Item	Answer	Frequency (N = 4)	Example of answer
Positive experience	Yes No	4 0	“For me, it was a positive experience. I felt it added depth to the sessions we had already done” (C1)
Negative experience	Yes No	1 3	“I did not feel prepared” (C1)
Experience of religiosity gap	Yes No	4 0	“The client experiences that very differently from me” (C4)
Application with other clients	Yes No	4 0	“If another client is interested, I would use it again” (C3)

All four clinicians were positive about the gained insights of the interview. For them it was a clarifying, in-depth and valuable conversation in which they learned new things about the clients and their faith experiences that they could use in their therapy. The most important thing of the FRI for the clinicians was the fact that religion and faith could be explicitly addressed during this conversation in a clearly structured way.

“For me, it was a positive experience. I felt it added depth to the sessions we had already done.” (C1)

All clinicians experienced a religiosity gap with all clients. However, since the FRI offers a common religious language with the goal to understand the client, the gap was for none of the clinicians a troublesome issue. The clinicians were positive about using the FRI with other clients, yet only if the client would be willing to integrate religion in their therapy. Half of the clinicians mentioned that they would like more practice to be able to use the FRI (C1, C3). Three clinicians would appreciate to have a group of colleagues to talk about the FRI and its report and ask for help if needed (C1, C2, C3). One clinician shared that she did not feel prepared enough because she was not present at the training (C1). This made her feel uncomfortable in the two interviews and resulted in the fact that the interviews took a long time. Correspondingly, the two clients of this clinician thought the interviews were taking too long. The same clinician also shared that she felt she was participating in this research, which made the FRI’s feel different.

### The Contribution in Terms of New Insights Regarding the FRI

#### New Insights Clients

The coding process highlighted several recurring insights for the clients (Table 5). Three of the clients reported that they had discussed faith-related topics with their clinicians before the FRI (P1, P6, P8). For one of them (P8) this topic had been frequently discussed, using a workbook on religious coping (Hoekzema-Kruidhof et al., 2021).

**Table 5:** Client Insights Attributed to the FRI

Item	Answer	Frequency (N = 8)	Example of answer
Faith in therapy before faith relationship interview	Yes No	3 5	“A while ago I used a workbook in therapy that focused on faith and emotions” (P8) “I have been in therapy for a while, but faith was never brought up” (P3)
Insight self-representation	Yes No	6 2	“My self-representation has expanded” (P4) “Not that I am aware of, no” (P1)
Insight God representation	Yes No	8 0	“The questionnaire gave me insight in my God representation” (P3)
Insight relationship others	Yes No	6 2	“I push people away, and I do that in relation to God as well. I suddenly recognized that” (P4) “I do not treat people around me the same as I treat God, because I believe God does not make mistakes” (P8)
Insight relationship faith and request for help	Yes No	7 1	“One of my core beliefs is that I am never good enough, and I experience that also in relation to God, because we are sinful people” (P5) “I don’t know, I don’t think so” (P1)
Insight in session after the faith relationship interview	Yes No	5 3	“I learned more in the session afterwards about the relationship between my faith and request for help than in the interview itself” (P8) “The session afterwards was just feedback” (P6)

All clients were asked whether the FRI gave new insights about themselves, their God representations, psychological struggles and their relationships with others. Two clients reported not to have learned something about themselves (P1, P2). One of them however shared later in the interview that she learned “why she thinks what she thinks” (P2). For the other client, the interview gave an insight in how she sometimes experiences God as absent (P1). Thus, implicitly both clients learned something about themselves. The other six clients learned something about their faith and how their representations of themselves are intertwined with their representations of God (P3-8). One client, for example, shared her new insight that she sees God as strict and therefore she is strict with herself as well (P7). For another client the FRI provided a new recognition of the relationship patterns she has formed because of her anxiety (P4). She shared that her self-representation has broadened by seeing those patterns in her relationship with God as well.

All clients reported to have learned something about their God representations; for five of them the FRI provided insights in the origin and development of their God representations (P1, P2, P3, P6, P7). Three others mentioned that the interview raised awareness of these representations (P4, P5, P8).

For six of the clients, the FRI has given an insight in the parallel between their relationship with others and their relationship with God (P2–P7). Four of them saw this as the greatest and most valuable insight of the FRI (P2, P4, P5, P7). They found out that they treat their relationship with God the same as their relationships with others. One client, for example, shared that she has a tendency to “push people away and to lock herself up,” and in the FRI she recognized that she does that in relation to God as well (P4).

Almost all clients confirmed that the FRI gave insight in the relationship between their faith and their request for help during therapy (P2-P8). For three of the clients, for example, their fear of not being good enough and their perfectionism was related to their faith as well (P4, P5, P8).

“One of my core beliefs is that I am never good enough, and I experience that also in relation to God, because we are sinful people” (P5).

On the question whether the clients learned something during the FRI or during the session with their clinician afterward, three of the clients reported to have only gained insights during the FRI. For them the session afterward was a summary of the FRI (P1, P2, P6). One of them, however, shared her positive experience during this session that she was able to read and discuss the report (P6). Two of the clients mostly gained insight during the session after the FRI (P5, P8) and three of the clients learned as much in the FRI as in the session afterward (P3, P4, P7).

### New Insights Clinicians

For all eight clients, the clinicians learned something new, even though the FRI’s took place during ongoing treatments (Table 6). For five clients of three different clinicians (C1, C2, C4), faith and religion were new topics in their therapy. Examples of insights reported by clinicians were on the experience and development of one client’s faith and on the reflection process of a client on her church culture.

“The message of the church contributes to her psychological struggles” (C4)

**Table 6:** Clinician Insights Attributed to the FRI

Item	Answer	Cl. 1, 2 FRI’s	Cl. 2, 2 FRI’s	Cl. 3, 1 FRI	Cl. 4, 3 FRI’s	Example of answer
Faith in therapy before faith relationship interview	Yes No	1 1	1 1	1 0	0 3	“No, we had never talked about religion before” (C2)
Insight God representation	Yes No	1 1	2 0	1 0	3 0	“I already knew this, because we had discussed it earlier” (C1)
Insight faith and request for help	Yes No	0 2	2 0	1 0	1 2	“The message of the church contributes to her psychological struggles” (C4)
Insight parallel relationship others and God	Yes No	2 0	2 0	0 1	3 0	“I definitely saw parallels and similarities, because she never felt good enough for people or for God” (C2) “No, the client was reluctant to talk about that” (C3)

*Cl.*, *clinician*

After most of the FRI’s the clinicians reported to have gained insights in the clients’ God representations (7) and in the parallels between the clients’ relationships with others and their relationship with God (7).

“I definitely saw parallels and similarities, because she never felt good enough for people or for God”(C2).

For half of the clients, the clinicians reported to have gained insights in the relationship between the clients’ faith and their request for help at Eleos.

### The Added Value Regarding the Integration of Religion in Therapy

The five clients who did not discuss religion in their therapy prior to the FRI would all like to integrate the topic of faith more in their therapy (P2, P3, P4, P5, P7). Although for some clients, faith should not be the main topic in therapy, they still would like to talk about it now and then (P2, P7). Other clients would like to integrate faith on a more regular basis (P3, P4, P5). One client for example shared that faith is part of her self-representation and God is part of her life, so she would appreciate to integrate that in therapy as well (P5). Of the three clients (P1, P6, P8) who did discuss religion in their therapy before the FRI, one of them does not want to integrate faith in further therapy sessions (P1). The other client almost finished her therapy at Eleos, which was the main reason she was not willing to discuss religion (P6). However, hypothetically, she would have appreciated to integrate faith more in her therapy if the FRI was conducted at the start of her therapy. The last client, who previously discussed religion in her therapy, is almost finished with her therapy as well (P8). However, she made an additional appointment with her clinician to specifically talk more about her faith. In conclusion, for six of the eight clients the FRI has influenced the role of faith in their therapy in a way that the clients wish to integrate the topic of faith more than they did before the FRI.

All clinicians were asked whether the therapy of their clients would change after and because of the FRI. For three of the clients, the clinicians did not consider changing the therapy. For the other five clients, the topic of faith would be discussed more by the clinicians.

## Discussion

The findings of this exploratory study suggest that both clients and clinicians view the FRI as a potentially valuable tool in therapy. According to the respondents, the FRI was positively received, with clients appreciating the structured approach and the clear opportunity to discuss their faith experiences. While the FRI did not change how most clients share their faith with others, it led to an expressed desire among both clients and clinicians to integrate faith more into therapy sessions.

These results correspond with previous research on the FRI (Mosterd-Pol et al., 2021), in which the FRI was experienced as a valuable method for depressive adults and with other research on religion and spirituality in therapy (Lee et al., 2019; Sandage et al., 2020; Van Nieuw Amerongen-Meeuse et al., 2020). In these studies, clients and clinicians experience the wish to integrate religion and spirituality in therapy and clients want to receive an explanation how religion plays a role in their mental struggles or discuss religious distress (Van Nieuw Amerongen-Meeuse et al., 2020). The FRI might be a method to put this wish into practice. Besides that, the FRI might fill the gap on “the interplay between God representations and attachment bonds” (Currier et al., 2021, p. 13).

Interestingly, although almost none of the clients experienced a religiosity gap, all clinicians reported to experience this gap. An explanation for this might be that the clinicians did not share a lot about their own positions regarding religion but tried to match closely to the experiences of the client. For the clinicians, however, the religiosity gap is not experienced as a problematic issue, because of the structure and language of the FRI and the goal to understand the clients. According to Van Nieuw Amerongen-Meeuse et al. (2018), in Christian mental healthcare institutions clients report positive experiences when there is a religiosity match or agreement regarding religion with their clinician. All clients felt comfortable regarding discussing religion and reported positive experiences. Therefore, the FRI might be a method to integrate religion, disregarding the differences in religious experiences between clients and clinicians.

In this research the choice has been made to include adolescent clients between 15 and 19 years old with different diagnoses. Although most research on religion and spirituality and mental health has been done among adults, research among adolescents has shown a positive relationship between religiosity and mental health (Cotton et al., 2006; Regnerus, 2003; Rew & Wong, 2006). The FRI might be an answer to the recommendations of previous research among adolescents to provide “clinical interventions that are sensitive to religious and spiritual values” (Dew et al., 2008, p. 250) and to develop interventions that incorporate religion in therapy for adolescents (Cotton et al., 2006). Because of the explorative nature of this research, all clients who were interested in participating were included. The clients were affiliated with different protestant denominations, which suggest that the FRI can be used among a broad Christian target group, regardless of the clients’ diagnoses.

Training seems to be an important preparation for the clinicians before conducting the FRI with their clients. This corresponds with research by Vieten et al. (2013) stating that religious and spiritual interventions should only be used in psychotherapy when the clinicians have had training, have knowledge about the literature and have clinical competence to use the intervention. For clinicians, it would be helpful to have a group of colleagues to talk about the FRI and the report and ask for help if needed. With more training and a group of colleagues, the clinicians would use the FRI with other clients, only if the clients would be willing to integrate religion in their therapy.

### Limitations

This research has an explorative nature and the evaluation interviews were based on self-designed questions. The current sample of eight clients and four clinicians can be seen as relatively small, and there is no control group. Furthermore, the participants were all female, both clients and clinicians. This was not an intentional selection criterion but rather a reflection of the natural sampling process, in which one clinician invited female colleagues, who in turn invited clients from their own caseloads. As a result, the sample emerged organically rather than through stratified or randomized recruitment. While this approach fits the exploratory and qualitative nature of the study, it does limit the representativeness of the findings. Gender may influence how religious and spiritual struggles are experienced and discussed in therapy, and male perspectives—whether as clients or clinicians—may differ significantly. Therefore, the results should be interpreted with caution and not generalized to broader or mixed-gender populations.

Furthermore, the FRI has been conducted by four different clinicians, which might have affected the results, even though they all had the same training. However, since different clinicians with different fields of expertise reported positive experiences regarding the FRI, it could be assumed that the FRI is widely applicable for different types of psychotherapy. Further research is needed to evaluate the effects of the FRI with different, wider samples, including, for example, male participants, validated measuring instruments and a control group. This research evaluates short-term opinions of clients and clinicians and effects of the FRI. More research is needed on long-term implications.

Alternative explanations can be mentioned for the results of this research. The FRI’s were conducted during ongoing treatments, with clients that were interested in integrating religion in their therapy. The clients might be more likely to rate the FRI’s positively, since a therapeutic alliance between client and clinician has been formed and the clients were open to discuss faith in their therapy. Besides that, the results can be affected by social desirability. The evaluation interviews by the researcher were conducted in one-on-one settings, and the participants might have shared answers they thought the researcher wanted to hear instead of their real thoughts. Further research that can avoid the social desirability bias is needed. The qualitative measurement strategy represents a strength of the study (Davis et al., 2016), in which the evaluation of the FRI has been studied for the second time with clients and for the first time with clinicians.

## Conclusion

Based on the experiences of the clients and clinicians in this study, the FRI seems to be a valuable intervention for exploring clients’ God representations, the parallels between their relationships with others and with God, and insights into the relationship between their requests for help and religious faith. Our findings suggest that the FRI may offer important insights into the role of religion and faith-related topics in therapy, although further research is needed to confirm its broader applicability and effectiveness across different client populations.
